# Small-Molecule Inhibitors of the Receptor Tyrosine Kinases: Promising Tools for Targeted Cancer Therapies

**DOI:** 10.3390/ijms150813768

**Published:** 2014-08-08

**Authors:** Mohammad Hojjat-Farsangi

**Affiliations:** Department of Oncology-Pathology, Immune and Gene Therapy Lab, Cancer Center Karolinska (CCK), Karolinska University Hospital Solna and Karolinska Institute, Stockholm 17176, Sweden; E-Mail: mohammad.hojat-farsangi@Ki.se; Tel.: +46-517-74308; Fax: +46-517-75897

**Keywords:** targeted cancer therapy, small-molecule inhibitors, receptor tyrosine kinases, tyrosine kinase inhibitors

## Abstract

Chemotherapeutic and cytotoxic drugs are widely used in the treatment of cancer. In spite of the improvements in the life quality of patients, their effectiveness is compromised by several disadvantages. This represents a demand for developing new effective strategies with focusing on tumor cells and minimum side effects. Targeted cancer therapies and personalized medicine have been defined as a new type of emerging treatments. Small molecule inhibitors (SMIs) are among the most effective drugs for targeted cancer therapy. The growing number of approved SMIs of receptor tyrosine kinases (RTKs) *i.e.*, tyrosine kinase inhibitors (TKIs) in the clinical oncology imply the increasing attention and application of these therapeutic tools. Most of the current approved RTK–TKIs in preclinical and clinical settings are multi-targeted inhibitors with several side effects. Only a few specific/selective RTK–TKIs have been developed for the treatment of cancer patients. Specific/selective RTK–TKIs have shown less deleterious effects compared to multi-targeted inhibitors. This review intends to highlight the importance of specific/selective TKIs for future development with less side effects and more manageable agents. This article provides an overview of: (1) the characteristics and function of RTKs and TKIs; (2) the recent advances in the improvement of specific/selective RTK–TKIs in preclinical or clinical settings; and (3) emerging RTKs for targeted cancer therapies by TKIs.

## 1. Introduction

Cancer is a complex disorder of the uncontrolled proliferation of cells. Currently, eight hallmarks explain the typical capabilities acquired by tumor cells in the process of tumorigenesis [1]. These properties are sustaining proliferation, evading growth suppressors, resisting cell death, enabling replicative immortality, activating invasion, metastasis, evading from the immune system, and reprogramming energy metabolism [1,2]. Recently, other features including epigenetic alterations have been introduced [3].

Multiple cytotoxic drugs and combination regimens have resulted in significant improvement of prognosis and survival of cancer patients. However, their therapeutic uses have been blunted by several disadvantages. This represents a request for developing more specific drugs.

Currently, rapid clinical application of targeted cancer therapy agents has dominated drug development. Targeted cancer therapy agents have significantly progressed during recent years by developing and receiving approval from authorities for use in cancer treatment. The aim of targeted cancer treatment is to destroy cancer cells by targeting tumor-specific (TSA) or -associated antigens (TAA) without affecting normal cells. In fact, tumor cells of different origins display unusual molecules that are either inappropriate for normal cells and their environment or are usually present during the organogenesis. For instance, receptor tyrosine kinase-like orphan receptor 1 (ROR1), a recently identified oncofetal protein, expresses in neuronal cells and other fetal tissues during embryogenesis. However, this receptor tyrosine kinase (RTK) has been found to be over-expressed in various tumors but not in normal adult cells [4,5,6,7,8,9].

For targeted cancer therapies, monoclonal antibodies (mAbs) and small-molecule inhibitors (SMIs) of tyrosine kinase activity (TKIs) are ideal candidates which target tumor cells via binding to cell-surface antigens and intracellular molecules, respectively [10].

An SMI is a type of compound that interferes with specific molecules required for tumor cell growth and function. SMIs selectively target molecules with a unique structure and are different from the non-specific destruction associated with traditional chemotherapy. They have been used for the treatment of a wide range of diseases, including infectious diseases, as well as autoimmune and malignant disorders.

Among the various targets for SMIs, receptor tyrosine kinases (RTKs) have attractive features. RTKs have distinct structural and biological properties that make them capable of transducing extracellular signals into the intracellular compartments [11]. Most oncogenic RTKs have no or low activity and expression in normal tissues, but are hyperactivated or upregulated in malignant cells [12]. Targeting RTKs by TKIs (RTK–TKIs) are under intensive research.

Most of the current RTK–TKIs are multi-targeted and inhibit a variety of molecules in a non-specific manner. Multi-targeted inhibition has been shown to have several disadvantages due to targeting various RTKs compared to selective/specific TKIs [13]. Only a few specific/selective TKIs have been approved by authorities for cancer treatment or are in preclinical and clinical settings.

In recent years, there has been a significant progress in developing specific/selective TKIs for targeted cancer therapy. Due to the lack of cumulative data on selective/specific TKIs, this review summarizes the recent findings regarding more specific/selective RTK–TKIs that are approved by authorities or are in preclinical and clinical settings. Moreover, approaching RTKs that might be proper candidates for targeted therapy will also be overviewed.

## 2. Receptor Tyrosine Kinases’ (RTKs’) Function and Structure

The term “oncogenic addiction” established by Weinstein [14] described that tumor cells may exhibit addiction on an activated oncogenic signaling pathway to sustain their survival and proliferation. Several oncoproteins, including tyrosine kinases, are known to be essential for the oncogenic process.

Protein kinases are enzymes that are involved in phosphorylation and transfer of a phosphate group from adenosine 3 phosphates (ATP) to tyrosine, serine or threonine residues. Protein phosphorylation is one of the most important events in regulating cell activities. Some oncoproteins need phosphorylation for regulation and activation [2].

Among different protein kinases, RTKs comprise a well-known group and consist of a transmembrane receptor linked to the intracellular kinase domain. These proteins have emerged as key pharmacological targets in oncology [15]. Phosphorylation of other RTKs, as well as intracellular intermediates by these kinases, is critical for signal transduction, regulation of cellular activity and function [16]. Among 58 known RTKs, 30 of them have been shown to be necessary for oncogenesis in various tumors [14].

Similar to transmembrane proteins, RTKs’ structure consists of three different parts: extracellular, transmembrane and cytoplasmic regions [17]. The extracellular part is preceded by a cleavable signal sequence and holds the binding sites that interact with ligands [18]. The extracellular domain is involved in the dimerization of RTKs, a process that is critical for the activation of intrinsic tyrosine kinase (TK) activity [19].

The cytoplasmic region contains tyrosine residues that are phosphorylated upon ligand binding and activation, regulate catalytic function, and also serve as docking sites for SRC Homology 2 (SH2) domain-containing proteins [20].

Deregulation of RTK activity is the major mechanism by which tumor cells escape from physiological constraints on survival and growth [2]. Aberrant RTK activation due to receptor over-expression, chromosomal translocation, gene amplification, mutations, and impaired receptor downregulation contribute to the development of various forms of cancer in human [21,22]. Some examples of these RTKs that are under investigation are listed in Table 1.

Identifying new oncogenic RTKs that are over-expressed by tumor cells and regulate the growth, survival, invasion, and communication of tumor cells with their microenvironment have facilitated the development of new anti-cancer agents and have revolutionized treatment options. Therefore, due to the interesting biological features, RTKs are of the main focus for developing new TKIs for therapeutic interventions in cancer patients.

## 3. Tyrosine Kinase Inhibitors (TKIs)

TKIs, as well as other small inhibitors, are low molecular weight organic compounds. A cut off at 500 Daltons is recommended based on the observation that clinical attrition rates are significantly reduced when the molecular weight falls below 500 Daltons (Table 2 and Table 3) [23,24]. The upper molecular weight is approximately 900 Daltons [25].

**Table 1:** Oncogenic receptor tyrosine kinases in cancer.

Oncogenic RTK (Examples)	Chromosome Location	Cancer (Examples)	Approved Selective TKI for Treatment
ALK	2p23	NSCLC, colorectal cancer, breast cancer	−
AXL	19q13.1	Lung, colon, breast, AML, CML	−
CCK4 (PTK7)	6p21.1	small cell lung cancer, breast cancer, gastric and colon cancer, AML	−
DDR1	6p21.33	NSCLC, breast cancer, AML, ovarian cancer	−
DDR2	1q23.3	NSCLC, lung cancers, CML, breast cancer	−
EGFR1 (ERBB1/HER1)	7p11.2	Breast cancer, hepatocellular carcinoma	+
EGFR2 (ERBB2/HER2)	17q12	Breast cancer, gastric adenocarcinomas	+
EGFR3 (ERBB3/HER3)	12q13.2	Breast cancer, ovarian cancer, Squamous cell lung cancer	+
EGFR4 (ERBB4/HER4)	2q34	Breast cancer, melanoma	+
EPHA1	7q35	NSCLC, prostate cancer	−
EPHA2	1p36.13	Hepatocellular carcinoma. colorectal cancer, breast cancer	−
EPHA3	3p11.1	Glioblastoma, lung cancer, melanoma, ALL	−
EPHA4	2q36.1	NSCLC, gastric cancer	−
EPHA5	4q13.1	Breast cancer, hepatocellular carcinoma, ALL	−
EPHB1	Xq13.1	NSCLC, cervical cancer, ovarian Cancer	−
EPHB2	13q33.3	Cervical cancer, breast cancer	−
EPHB3	3q27.1	NSCLC, breast cancer, colorectal cancer	−
EPHB4	7q22.1	Breast cancer, melanoma, glioma	−
FGFR1	8p12	Squamous cell lung cancer, breast cancer	−
FGFR2	10q26	Squamous cell lung cancer, breast cancer, thyroid cancer	−
FGFR3	4p16.3	Bladder cancer, squamous cell carcinoma	−
FLT3	13q12.2	AML, acute promyelocytic leukemia	−
IGF1R	15q26.3	CLL, breast cancer, pancreatic cancer	−
IGF2R	6q25.3	breast cancer, prostate cancer, colorectal carcinoma	−
INSR	19p13.2	Colorectal cancer, prostate cancer	−
INSRR	1q23.1	Neuroblastoma	−
KIT	4q12	AML, melanoma, ovarian carcinoma	−
LTK	15q15.1	Gastric cancer, lymphomas and leukemias	−
MER	2q13	Glioblastoma, hepatocellular carcinoma	−
MET	7q31.2	Hepatocellular carcinoma, CLL, breast cancer	−
MUSK	9q31.3	Ovarian cancer	−
NTRK1 (TrkA)	1q21-22	Colorectal cancer, breast cancer	−
NTRK2 (TrkB)	9q22.1	Neuroblastoma, astrocytoma	−
NTRK3 (TrkC)	15q25	Neuroblastoma, breast cancer	−
PDGFRA	4q12	Lung adenocarcinoma, gastrointestinal stromal tumors	−
PDGFRB	5q32	gastrointestinal stromal tumors, glioblastoma	−
RET	10q11.2	NSCLC, medullary thyroid carcinoma	−
RON (MST1R)	3p21.31	Pancreatic cancer, breast cancer, NSCLC	−
ROR1	1p31.3	CLL, ALL, AML, MCL, HCL, melanoma	−
ROR2	9q22.31	Melanoma, hepatocellular carcinoma, colon cancer	−
ROS1	6q22	NSCLC, ovarian cancer	−
RYK	3q22.2	CML, ovarian cancer	−
TIE	1p34.2	Glioblastoma, breast tumor	−
TEK	9p21.2	Bladder cancer, glioblastoma, AML	−
TYRO3	15q15.1	Colon cancer, melanoma, thyroid cancer, breast cancer	−
VEGFR1 (FLT1)	13q12.3	Ovarian cancer, NSCLC, colorectal carcinoma	+
VEGFR2 (KDR)	4q12	Renal cell carcinoma, breast cancer	+
VEGFR3 (FLT4)	5q35.3	Thyroid carcinoma, breast cancer	+

ALK: anaplastic lymphoma receptor tyrosine kinase, NSCLC: non-small cells lung carcinoma, AML: acute myeloid leukemia, CML: chronic myeloid leukemia, DDR: Discoidin domain receptor, EGFR: epidermal growth factor receptor, EPHA: ephrin type-A receptor, ALL: acute lymphoid leukemia, EPHB: ephrin type-B receptor, FGFR: fibroblast growth factor receptor, FLT3: Fms-like tyrosine kinase 3, IGFR: insulin growth factor receptor, CLL: chronic lymphocytic leukemia, INSR: insulin receptor, LTK: leukocyte tyrosine kinase, NTRK: neurotrophic tyrosine kinase, PDGFR: platelet-derived growth factor receptor, ROR: receptor tyrosine kinase-like orphan receptor, VEGFR: vascular endothelial growth factor receptor.

**Table 2:** Current specific/selective tyrosine kinase inhibitors (TKIs) targeting receptor tyrosine kinases (RTKs).

Name	Trade/Code Name	Mol. Mass (g/mol)	Selective Target	IC_50_ (nM/L) *	FDA Approved	Cancer (Examples)
Afatinib	Gilotrif	485.94	HER2, EGFR	0.5, 14	+	NSCLC, squamous cell carcinoma of the head and neck, breast cancer
Canertinib	CI-1033	485.94	EGFR, HER2, 4	0.8,19, 7	−	Head and neck, breast, and NSCLC, ovarian cancer
Cediranib	Recentin	450.5	VEGFRs	<1	−	NSCLC, kidney and colorectal cancer
CP-673451	–	417.5	PDGFRs	<1	−	NSCLC, colon carcinomas, glioblastoma
Crizotinib	Xalkori	450.34	MET	11	+	NSCLC, anaplastic large cell lymphoma, neuroblastoma
Crenolanib	CP-868-596	443.54	MET, ALK, FLT3, PDGFRα,β	11, 24, 0.74, 1, 0.4	−	AML, gastrointestinal stromal tumor, glioma
Dacomitinib	PF-00299804	469.94	EGFR	6	−	NSCLC, gastric, head and neck cancer, glioma
Erlotinib	Tarceva	393.43	EGFR	2	+	NSCLC, pancreatic cancer
EMD1214063	–	492.57	MET	3	−	NSCLC
EMD1204831	–	–	MET	9	−	NSCLC
Gefitinib	Iressa	446.9	EGFR	<57	+	NSCLC, AML
Icotinib	Conmana	391.15	EGFR	5	+	NSCLC
KW-2449	–	332.4	FLT3	6.6	−	AML
Lapatinib	Tykerb	581.06	HER-2, EGFR	9.2, 10.8	+	Breast cancer
Lenvatinib	E7080	426.85	VEGFR2, 3	<4	+	Approved for thyroid cancer in Japan
LY2801653	–	552.53	Met, RON	<2	−	NSCLC
Neratinib	HKI-272	557.04	EGFR, HER2	92, 59	−	NSCLC, breast cancer
PD-173074	–	523.67	FGFRs	<25	−	NSCLC, gastric carcinoma, breast cancer
Quizartinib	AC220	560.67	FLT3	<4.2	−	AML
R428	BGB-324	506.64	AXL	14	−	AML, NSCLC, breast cancer
Tandutinib	MLN518/CT53518	562.7	FLT3	<100	−	RCC, CML
Tivantinib	Arqule/ARQ-197	369.42	MET	4	−	RCC, breast cancer
Tivozanib	AV-951	454.86	VEGFR1, 2, 3	0.21, 0.16, 0.24	−	RCC, breast cancer
Vatalanib	PTK787/ PTK/ZK	346.81	VEGFR2	37	−	NSCLC, DLBCL, colorectal adenocarcinoma

* Half maximal inhibitory concentration (IC_50_) values are the measure of the effectiveness of TKIs in inhibiting the RTKs in biochemical assays, HER: human epidermal receptor, EGFR: epidermal growth factor receptor, NSCLC: non-small cells lung carcinoma, VEGFR: vascular endothelial growth factor receptor, PDGFR: platelet-derived growth factor receptor, ALK: anaplastic lymphoma receptor tyrosine kinase, FLT3: Fms-like tyrosine kinase 3, AML: acute myeloid leukemia, CML: chronic myeloid leukemia, RCC: renal cell carcinoma, DLBCL; Diffused large B-cell lymphoma.

**Table 3:** Multi-targeted tyrosine kinase inhibitors (TKIs) targeting RTKs and intracellular kinases.

Name	Trade/Code Name	Mol. Mass (g/mol)	Target Molecules (Examples)	IC_50_ (nM/L) *	FDA Approved	Cancer (Examples)
Amuvatinib	MP470	447.51	ALK, MER, KIT, RET, PDGFRs, FLT3, RAD 51	<100	−	NSCLC
Axitinib	Inlyta	386.5	VEGFRs, PDGFRs, KIT	<1.7	+	RCC
Cabozantinib (XL184)	Cometriq	501.51	VEGF, RET, MET, NTRKB, TIE2, AXL	<15	+	Medullary thyroid cancer, progressive metastatic medullary thyroid cancer
Dasatinib	Sprycel	488.01	BCR-ABL, SRC, KIT, PDGFRs, EPH, CSK	<10	+	CML, ALL
Foretinib	–	632.65	VEGFR2, MET	0.9, 0.4	−	NSCLC, breast, gastric, papillary renal cancer
Golvatinib	E7050	633.69	VEGFR2, MET	16, 14	−	Gastric cancer, HCC, glioblastoma, melanoma
Imatinib	Gleevec	589.7	ABL, KIT, PDGFRs	0.6, 0.1, 0.1	+	Gastrointestinal stromal tumor, leukemias
MGCD-265	–	517.6	MET, VEGFRs, TIE2, RON	<7	−	NSCLC
Nilotinib	Tasigna	529.5	BCR-ABL, KIT, LCK, EPHA3, 8, DDR1, 2	<30	+	CML
Pazopanib	Votrient	437.51	PDGFRs, VEGFRs	<150	+	Advanced renal cell carcinoma, advanced soft tissue sarcoma
Ponatinib	Iclusig	532.56	BCR-ABL, PDGFRα, SRC, KIT, FGFR, VEGFRs	<6	+	CML, philadelphia chromosome positive ALL
Regorafenib	Stivarga	482.82	TIE2, PDGFRs, RET, KIT, B-RAF	<25	+	Metastatic colon cancer
Sorafenib	Nexavar	464.8	VEGFRs, PDGFRs, B-RAF, MEK, ERK	<100	+	Advanced renal cell carcinoma, hepatocellular carcinoma
Sunitinib	Sutent	532.56	VEGFR2, PDGFRβ, KIT, RET, CSF1R, FLT3	<100	+	Renal cell carcinoma, gastrointestinal stromal tumor
Vandetanib	Caprelsa	475.35	EGFR, VEGFRs, RET, Tie-2, FGFR1	<500	+	Metastatic medullary thyroid cancer

* Half maximal inhibitory concentration (IC_50_) values are the measure of the effectiveness of TKIs in inhibiting the RTKs in biochemical assays, ALK: anaplastic lymphoma receptor tyrosine kinase, FLT3: Fms-like tyrosine kinase 3, PDGFR: platelet derived growth factor receptor, EGFR: epidermal growth factor receptor, NSCLC: non-small cells lung carcinoma, VEGFR: vascular endothelial growth factor receptor, VEGF: vascular endothelial growth factor, NTRK: neurotrophic tyrosine kinase, EPHA: ephrin type-A receptor, DDR: Discoidin domain receptor, CML: chronic myeloid leukemia.

Proper TKIs are usually selected by high-throughput screening (HTS) methods that detect the most proper TKI candidates among a large library of compounds. An optimal TKI should have particular characteristics for further development. Absorption, distribution, metabolism, excretion, and toxicity (ADMET) of a drug candidate are the most important elements that should optimize for *in vivo* use [26]. Based on these properties, there are several challenges in front of the selection of effective inhibitors. Membrane permeability, inactivation due to metabolism of the drug, decreasing due to non-specific interactions with other intracellular components or rapid excretion, toxicity and lack of distribution into appropriate cellular compartment are the major problems behind TKI discovery [26].

**Figure 1:**
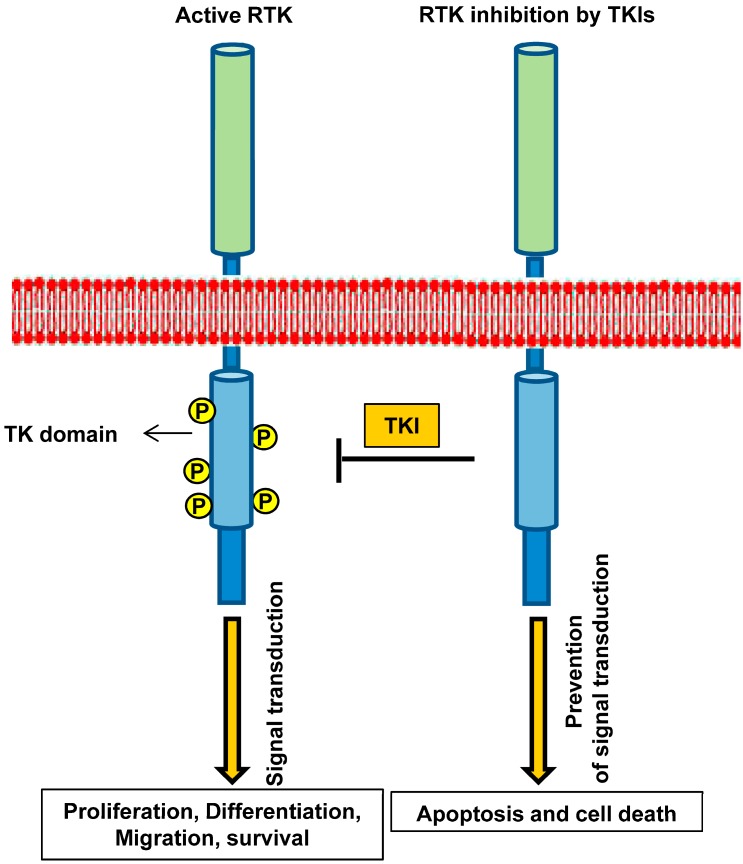
Targeting receptor tyrosine kinases by tyrosine kinase inhibitors (TKIs). Blocking small molecule inhibitors of kinase domain (TKIs) prevents the phosphorylation of the receptor at TK domain and interferes with cell proliferation, differentiation, migration, and survival and induces cell apoptosis. Phosphate groups are denoted as yellow circles.

TKIs prevent and block vital pathways through targeting signaling molecules which are necessary for cell survival. TKIs can translocate through the plasma membrane and by interacting with the cytoplasmic domain of RTKs and inhibit the catalytic activity of the TK domain by interfering with the binding of ATP or its substrates (Figure 1) [27].

TKIs are classified into three main groups. Most of the current TKIs are ATP-competitive inhibitors and are classified as type I inhibitors. Due to the highly conservative ATP-binding sites in TK domains and a high rate of competition with intracellular ATP, several difficulties obstruct the development of specific/selective TKIs of type I. Therefore, TKIs might target other kinases, thereby suggesting that the anti-tumor effects may be due to the effects on other signaling molecules. Types II and III are non-ATP competitors and act through induction of structural changes in the RTKs. The conformational shifts modify the TK domain in a way that the TK domain loses its kinase activity [28]. Moreover, these inhibitors can bind to residues within the TK domain and prevent tyrosine phosphorylation. Most of the TKIs that have been described in this review are type I inhibitors (Table 2 and Table 3).

## 4. Specific/Selective TKIs Targeting RTKs

Most of the FDA approved TKIs for the treatment of cancer are multi-targeted inhibitors of several intracellular tyrosine kinases (Table 3), and a few specifically inhibit the members of a family. Here, the most specific/selective TKIs that target the members of a specific RTK family are discussed (Table 2).

## 5. Epithelial Growth Factor Receptor (EGFR) Family and Specific/Selective TKIs

EGFR (ErbB) is a family of four structurally related RTKs: ErbB-1/ EGFR, ErbB-2/HER2/neu, ErbB-3/HER3, and ErbB-4/HER4. This family plays critical roles in the regulation of normal cell proliferation, differentiation and survival. Under physiologic circumstances, specific soluble ligand (EGF) binds to the extracellular region of EGFR and following homo/heterodimerization with other members lead to phosphorylation at specific tyrosine residues within the intracellular domain [29].

EGFR members are abnormally activated by several mechanisms like receptor over-expression, mutation, ligand-dependent receptor dimerization, ligand-independent stimulation, and are associated with the development of tumors of epithelial origin, including non-small cell lung cancer (NSCLC) [30], breast [31], colorectal [32], and pancreatic cancer [33]. Moreover, EGFR expression has been shown to be associated with a poor prognosis in most malignancies [34,35]. Therefore, specific/selective inhibition of EGFR is an ideal approach to cancer treatment.

### 5.1. Gefitinib and Erlotinib

Gefitinib (ZD1839, Iressa) [36] and erlotinib (OSI-774, Tarceva) [37] belong to the first generation of TKIs and are selective EGFR–TKIs that were approved on May 2003 and November 2004 for the treatment of NSCLC patients, respectively (Table 2) [30,38].

Erlotinib has also been approved for the treatment of patients with metastatic pancreatic cancer in combination with gemcitabine (2 November 2005) [39]. Anti-tumor effects of gefitinib and erlotinib have been investigated in other EGFR^+^ tumors, including gastric [40], gastroesophageal, esophageal [41], cervical [42], renal cell carcinoma [43], and hepatocellular carcinoma [44]. With a few exceptions, most trials have failed to show potent clinical effects in the majority of patients. Erlotinib has been shown to be effective as first-line treatment in gastroesophageal cancer, but it has shown no clinical benefits in gastric cancer [40].

There are several reviews on the preclinical and clinical studies of these two EGFR–TKIs and will therefore not be described more in this article [45,46].

### 5.2. Icotinib Hydrochloride

Icotinib hydrochloride (BPI-2009H) is a specific/selective EGFR–TKI that is approved for the treatment of NSCLC patients in China (Table 2). This TKI was developed in China and has similar structure, mechanism and therapeutic effects to gefitinib and erlotinib [47]. Icotinib has emerged as a promising TKI with clinical effects for the treatment of NSCLC patients [47].

Preclinical studies have demonstrated significant activity of icotinib on tumor cells. Icotinib blocks EGFR phosphorylation (IC_50_ = 45 nM) in A431 cell line and inhibits tumor cell proliferation. *In vivo* studies showed that Icotinib has dose-dependent anti-tumor effects in nude mice carrying human tumor-derived xenografts. The drug was well tolerated at doses up to 120 mg/kg/day in mice [48].

Pharmacokinetic analysis has shown that the mean half-life of icotinib was 6 h. The most frequent side effects were acnei-form dermatitis, diarrhea and a decrease in white blood cells [49].

Currently, several phase I, II and III clinical trials are ongoing in patients with NSCLC and head and neck squamous cell carcinoma [48,50].

### 5.3. Afatinib and Neratinib

Afatinib (Gilotrif) and neratinib (HKI-272) are the second generation of EGFR–TKIs that were studied in clinical trials either as monotherapy or in combination with other drugs (Table 2) [30].

These EGFR–TKIs are dual-inhibitors of EGFR and HER2. They bind to the EGFR and HER2 receptors and reduce autophosphorylation in cells by targeting a cysteine residue (Cys) in the ATP-binding pocket of the receptors.

The activity of gefitinib and erlotinib has been shown to be impressive in patients with EGFR mutations. However, most patients have developed acquired resistance to these TKIs. To overcome the problem, afatinib was developed as an irreversible EGFR–TKI.

Afatinib has been investigated as a first-line treatment for patients with EGFR mutation-driven NSCLC and as a second- or third-line treatment in patients with NSCLC, who have received prior treatment with an EGFR–TKI [51]. It has been shown that afatinib is an irreversible inhibitor of HER4 and covalently binds to the cysteine in position 803 [52].

Preclinical studies have shown the promising activity of neratinib in NSCLC and breast cancer. Cell lines transformed with HER2 have shown to be resistant to the erlotinib; however, these cells as well as NSCLC cell line, Calu-3, over-expressing HER2, were highly sensitive to the neratinib [53]. Moreover, HER2 over-expressing BT474 cells xenografts also showed high sensitivity to the neratinib [54]. Currently, neratinib is under investigation as monotherapy or in combination with other drugs in clinical trials (ClinicalTrials.gov).

### 5.4. Lapatinib

Lapatinib (GW572016, Tykerb) is a reversible inhibitor of EGFR/HER2 and was developed in 2002 for dual-targeted therapies (Table 2) [55]. Lapatinib binds covalently to the Cys 773 of EGFR and Cys 805 of HER2 with increased efficacy against mutant cell lines resistant to erlotinib and gefitinib [56]. It inhibits EGFR and HER2 tyrosine phosphorylation at the TK domain and prevents the activation of ERK1/2 and AKT followed by apoptosis of tumor cells *in vitro* and *in vivo* in xenografted mice with cell lines over-expressing EGFR and HER2 [55,57]. In the first preclinical studies of lapatinib, the effects were evaluated in human cell lines over-expressing EGFR or HER2 including HN5 (head and neck), A-431 (vulva), BT474 (breast), CaLu-3 (lung), and, N87 (gastric) cell lines. Lapatinib showed inhibition of cell growth following dephosphorylation of EGFR, HER2, AKT, and inhibited tumor xenograft growth of the HN5 and BT474 cells in mice [57].

Phase I clinical studies started at 2002 and the safety, tolerability and pharmacokinetics were assessed in healthy controls [58]. Biological effects on tumor cells were studied in patients with metastatic cancers over-expressing HER2 and EGFR [59]. Data showed variable effects on downregulation of ERK and AKT from biopsies of treated patients and few side effects as well as some evidence of clinical activity.

In combination with capecitabine, lapatinib has shown anti-tumor activity and prolongation of the time to progression of the disease in breast cancer patients who have previously been treated with trastuzumab, an anthracycline and a taxane [60]. Lapatinib was approved by the FDA on 13 March 2007 as the second-line treatment of breast cancer. Lapatinib was also approved as first-line therapy for treatment of postmenopausal women with estrogen/HER2 receptor-positive breast cancer in combination with an aromatase inhibitor.

### 5.5. Canertinib

Canertinib (PD183805/CI-1033) is an irreversible EGFR–TKI with activity against EGFR. It has shown anti-tumor activity against A341 and H125 cell lines *in vivo* in xenografted mice. It has been tested in phase I trials in patients with head and neck, breast and NSCLC cancer. The water-soluble analog of canertinib has been shown to inhibit EGFR activity *in vitro* and suppressed human A431 cells xenografts in nude mice [61].

Canertinib has been demonstrated to increase the anti-proliferative effects of vemurafenib in the BRAF mutant melanoma cell lines, but little or no enhanced effect was noted with the combination treatment in the wild type melanoma cell lines [62].

Canertinib decreased the phosphorylation of an ErbB kinase signaling target p70S6-kinase T389 in a dose-dependent manner as well as inactivation of downstream signaling molecules in ALL cell lines. Canertinib also increased the expression of the pro-apoptotic protein BIM, caspase-3 cleavage followed by apoptosis, abrogated proliferation and increased sensitivity to BCR/ABL-directed TKIs [63].

Several clinical trials are testing the anti-tumor activity of canertinib in metastatic breast cancer [64], NSCLC [65] and advanced ovarian cancer [66].

Currently, a few other selective EGFR–TKIs, including PKI-166, are under investigation in preclinical and clinical settings [67].

### 5.6. Dacomitinib

Dacomitinib (PF-00299804, PF299) is a selective, quinazalone-based irreversible pan-EGFR–TKI and is a proper candidate for the treatment of NSCLC (Table 2). Preclinical studies have shown that it inhibited the kinase activity of wild-type EGFR similar to gefitinib, erlotinib and canertinib. In contrast to gefitinib and erlotinib, it also inhibits HER2 and HER4 activity [68]. LCK and SRC were the only other kinases inhibited by dacomitinib, but the IC_50_ is >10-fold higher than that against EGFR [69]. *In vitro* assays have shown that dacomitinib is active in gefitinib-sensitive and gefitinib-resistant cells as well as EGFR and HER2 mutated NSCLC cell lines with or without MET amplification. Dacomitinib inhibited EGFR phosphorylation and downstream signaling molecules, including AKT and ERK1/2, and induced apoptosis in the EGFR T790M-containing H3255 GR cell line at 10 nmol/L concentration.

In xenografted nude mice using HCC827 GFP and HCC827 Del/T790M cells, either gefitinib or dacomitinib effectively inhibited the growth of HCC827 GFP xenografts. However, HCC827 Del/T790M xenografts were resistant to gefitinib, whereas dacomitinib was more effective at inhibiting growth of this xenograft model [69].

Dacomitinib irreversibly inhibited EGFR autophosphorylation in the A431 squamous cell carcinoma cell line with IC_50_ of 15.1 nmol/L alone or in the presence of EGF. In contrast, the reversible EGFR inhibitor erlotinib or gefitinib did not show the same inhibitory activity [70].

This TKI effectively prevented tumor growth in H125, SKOV3 and A431 cells of xenografted mice models and its therapeutic activity ranged from delayed progression to complete regressions [70]. SKOV3 xenografts, over-expressing HER2, EGFR, HER3, and HER4 exhibited an average tumor growth delay that ranged from 17.1 days (15 mg/kg) to 41.2 days (30 mg/kg) after treatment with dacomitinib and complete tumor regressions at doses as low as 15 mg/kg. At the maximum tolerated dose of 30 mg/kg, there were six of six complete tumor regressions seen with dacomitinib. HER2 phosphorylation at tyrosine residue (Tyr) 1248 was also inhibited with an average of 67%, 99% and 65% at 6, 24 and 48 h after dosing with 30 mg/kg of dacomitinib, respectively. In experiments using the A431 human squamous cell carcinoma xenografted model which over-expresses EGFR, HER2 and HER3, the anti-tumor effects were seen with an average tumor growth delay of 21 days at doses as low as 1.2 mg/kg [70].

Moreover, dacomitinib showed significant effects *in vitro* and *in vivo* against NCI-H1975 cells containing the EGFR L858R, T790M mutation. These preclinical models suggest that dacomitinib may be quite effective against lung cancer that becomes resistant to gefitinib or erlotinib via acquisition of a T790M mutation in EGFR [70].

The preliminary data of a phase II clinical trial as a first-line agent in squamous cell carcinoma of the head and neck showed a median progression-free survival (PFS) of 2.8 months and overall survival (OS) of 8.3 months. The most common side effects were diarrhea (16%), fatigue (9%), acnei-form dermatitis (7%), and hand–foot reaction (4%) [68].

A phase II study compared dacomitinib with erlotinib as second- or third-line therapy for patients with NSCLC [68]. The median PFS was 2.86 months in the dacomitinib arm and 1.91 months in the erlotinib arm (hazard ratio (HR) = 0.66; 95% confidence interval (CI) = 0.47–0.91; *p* = 0.012). Regarding the subgroup of patients with EGFR mutations, 19 patients received dacomitinib and 11 received erlotinib. The PFS (median) was 7.44 months in both arms (HR = 0.46; 95% CI = 0.18–1.18; *p* = 0.098). The most side effects in the dacomitinib arm were diarrhea and acnei-form dermatitis [68]. Based on the results of this study, a phase III trial (ARCHER study) of second- or third-line dacomitinib *versus* erlotinib has been started in patients with NSCLC and KRAS wild-type NSCLC (ClinicalTrials.gov, NCT01360554) [69].

Currently, a phase II trial to evaluate dacomitinib in first-line treatment for advanced lung adenocarcinoma is ongoing in patients with EGFR mutations (ClinicalTrials.gov, NCT00818441) [71]. Dacomitinib has also advanced to several phase III clinical trials.

## 6. Vascular Endothelial Growth Factor Receptor (VEGFR) and Anti-Angiogenesis TKIs

The VEGF family (VEGF-A (usually referred to VEGF), VEGF-B, VEGF-C, VEGF-D, and placental growth factor (PGF)) are over-expressed by various solid tumors and bind to its receptors (VEGFRs: VEGFR1, VEGFR2 and VEGFR3) on the vascular endothelium and induce angiogenesis. Angiogenesis is a regulated process responsible for the development of new blood vessels from a pre-existing vascular network.

At present, various selective VEGFR–TKIs, including vatalanib, tivozanib, cediranib, and lenvatinib, are under investigation for the treatment of various solid tumors.

### 6.1. Vatalanib

Vatalanib (PTK787 or ZK222584) has been shown to inhibit tumor angiogenesis and has been studied for the treatment for several types of cancer (Table 2) [72]. It selectively inhibited all VEGF receptors, but also inhibited other class III kinases, including platelet-derived growth factor receptor (PDGFR) β and KIT in higher concentrations. Several studies have shown that vatalanib induced apoptosis of CLL cells both *in vitro* and *in vivo* [73].

Preclinical investigations have shown that vatalanib inhibited growth and reduced microvasculature in subcutaneously implanted human tumor xenografts in nude mice [74]. It inhibited VEGF-induced autophosphorylation of VEGFRs, endothelial cell proliferation, migration, invasion, and survival *in vitro*. In concentrations up to 1 μM, vatalanib did not have any cytotoxic or anti-proliferative effect on cells that do not express VEGFR. It induced dose-dependent inhibition of VEGF-induced angiogenesis in a growth factor implanted and a tumor cell-driven angiogenesis model. Moreover, it inhibited the growth of various human carcinomas in nude mice as well as a murine renal carcinoma and its metastases in a syngeneic, orthotopic model. Microvessel formation has revealed to be inhibited by vatalanib in the interior of the tumor [74].

Anti-tumor and anti-angiogenic activity of vatalanib have been demonstrated in a murine renal cell carcinoma model. Treatment of mice with vatalanib showed no changes in extravasation, whereas a significant decrease in vessel permeability was noted. Moreover, an increase in partial blood volume was observed in the vatalanib-treated mice, although vessel density was reduced. Reduction in vessel density was shown to be due to the loss of microvessels [75].

Dual-inhibition of VEGF by prevention of VEGF production and VEGFR signaling has shown synergistic anti-tumor effects. *In vitro* effects of vatalanib and everolimus on cell proliferation, cell cycle, apoptosis, and signal transduction have been investigated in gastric cancer cell lines [76]. Results showed that everolimus but not vatalanib decreased gastric cancer cell proliferation with no effects on apoptosis. Vatalanib eliminated endothelial cell tube formation, but it was incomplete by everolimus. *In vivo* analysis showed that the combination of vatalanib and everolimus was more effective than single agent treatments and it reduced tumor size *in vivo*. Moreover, lower vascular density and proliferation was observed for combination treatment. Current data showed that vatalanib anti-tumor activity may be augmented in combination with other drugs including everolimus [76].

### 6.2. Tivozanib

Tivozanib (AV-951, KRN-951) is an oral quinoline urea derivative that suppresses angiogenesis by being selectively inhibitory against the VEGFR family. Although it might target other kinases with high concentration, it is highly specific for VEGFR with picomolar potency to each VEGFR member (Table 2).

Tivozanib has shown anti-tumor effects in human breast, colon, liver, lung, ovarian, pancreas, and prostate cancer and in brain xenografted models [77]. Tivozanib inhibited VEGF-induced VEGFR2 phosphorylation in endothelial cells at *in vitro* and inhibited VEGF-mediated migration of human umbilical vein endothelial cells. Athymic rats treated with tivozanib have been shown to decrease the microvessel density within xenografts. Tivozanib attenuated VEGFR2 phosphorylation in tumor endothelium and displayed anti-tumor activity against a wide variety of human tumor xenografts. Dynamic contrast-enhanced magnetic resonance imaging has revealed reduction in tumor vascular hyper-permeability that is associated with the tivozanib anti-tumor activity [78]. The effects have been evaluated in a rat colon cancer RCN-9 syngeneic model in which the tumor cells have been transplanted into the peritoneal cavity of F344 rats. Treatment of RCN-9 transplanted mice decreased angiogenesis, the formation of tumor nodules and the accumulation of malignant ascites. Furthermore, this TKI displayed regression of vascularization in newly formed tumor and increased the survival of rats even with more advanced-stage tumors [79].

Several clinical trials in phases II and III have shown benefits for patients with renal cell carcinoma [80] and breast cancer [81], and other clinical trials are ongoing for the treatment of different cancers using tivozanib alone or in combination [77,82].

### 6.3. Cediranib

Cediranib (Recentin, AZD2171) is an indole-ether quinazoline that is a highly potent VEGF-signaling inhibitor and IC_50_ value of <1 nmol/L *in vitro* (Table 2) [83]. In human endothelial cells, cediranib has been shown to inhibit VEGF-induced phosphorylation of VEGFR2 and VEGFR3 at concentrations of less than one nmol/L. It inhibited activation of downstream signaling molecules, including AKT, ERK1/2 and CREB. Cediranib also inhibited VEGFR3-mediated endothelial cell function and lymphangiogenesis, VEGF-induced proliferation, survival and migration of vascular endothelial cells followed by angiogenesis. It also inhibited lymphangiogenesis in NMRI nu/nu mice [83].

In a pilot study, combined imaging, microcomputed tomography and histologic tumor evaluation with a xenografted model of breast cancer cell line, cediranib induced significant prevention of tumor growth and regression of MCF7–VEGF cells (transfected with VEGF) in xenografted athymic mice. Moreover, blood flow and microvessel density and proliferation have been shown to decrease significantly in MCF7–VEGF tumors [84].

Several preclinical studies demonstrated the significant reduction of tumor growth in various cancers including kidney, breast, lung, prostate, ovarian, colorectal cancer, sarcomas, glioblastoma, renal cell carcinoma, and NSCLC [85]. Based on the promising results, several clinical trials have been started from 2007. However, most phase III clinical trials failed to show significant benefits from treatment with cediranib as monotherapy or in combination in NSCLC, glioblastoma, kidney, and colorectal cancer [86,87].

## 7. Platelet-Derived Growth Factor Receptor (PDGFR) and Specific/Selective TKIs

PDGFRs belong to the type III tyrosine kinase family. They are not expressed in normal tissues but only in fibroblasts, smooth muscle cells in lungs and pericytes of the vascular wall. PDGFR over-expression leading to constitutive PDGFR activation has been reported in a number of malignancies, including NSCLC and gliomas [88]. PDGFRs stimulate MAPK, PI3K and PLC-γ signaling pathways that are involved in multiple cellular and developmental responses [89].

All approved PDGFR–TKIs are multi-targeted and a few specific/selective inhibitors are in preclinical and clinical settings.

### 7.1. CP-673451

CP-673451 is a highly selective benzimidazole, reversible and ATP-competitive inhibitor of PDGFR. This TKI is 450–5000-fold more selective for PDGFRβ compared with other angiogenic RTKs [90]. *In vitro* experiments have demonstrated that CP-673451 inhibited cell growth of mesenchymal-like NSCLC cell line H1703 with high expression of PDGFRα, but not in the epithelial cell line H292 lacking PDGFR [91].

The anti-tumor efficacy has been investigated in tumor xenografted athymic mice, including lung and colon carcinomas and glioblastoma. This TKI inhibited PDGFR phosphorylation, PDGF-BB-stimulated angiogenesis *in vivo* and causes significant tumor growth inhibition in xenografted models [90].

CP-673451 decreased cell proliferation through dephosphorylation of PDGFR, AKT, GSK-3α, GSK-3β, and impaired rhabdosphere-forming capacity in both RD and RUCH2 rhabdomyosarcoma cells [92]. It decreased proliferation, tumor growth and stromal cell infiltration in xenografted mice with RD and RUCH2 cell lines with high expression of PDGFR, whereas no effects were observed in PDGFR negative cell line RMS [92].

CP-673451 has shown proper and selective inhibition of PDGFR in preclinical settings, but more investigations on various PDGFR over-expressing tumors are necessary.

### 7.2. Crenolanib

Crenolanib (CP-868596) is a selective inhibitor of PDGFR. It is 100-fold more selective for PDGFR than for other kinases like VEGFR2 and fibroblast growth factor receptor (FGFR) 2 [93].

Crenolanib has been evaluated in a phase I, open-label, dose-escalation study designed to evaluate the safety, tolerability and pharmacokinetics. A dose of 100 mg twice daily with food was shown to be well tolerated [93].

The activity of crenolanib has been compared with imatinib in a panel of mutated PDGFRα expressing cell lines [88]. Crenolanib displayed strong anti-proliferative activity in BaF3 D842V and EOL-1 cell lines with PDGFRα-dependent growth. Data indicated that crenolanib was significantly more potent than imatinib in inhibiting the kinase activity of imatinib-resistant mutated PDGFRα including D842I, D842V, D842Y, DI842-843IM, and deletion I843. It showed to be 135-fold more potent than imatinib against primary gastrointestinal stromal tumors cells expressing PDGFRα with D842V deletion and IC_50_ of approximately 10 nmol/L [88].

## 8. Fibroblast Growth Factor Receptor (FGFR)

The FGFR family includes four members (FGFR1-4). Upon ligand binding, FGFRs activate mainly the mitogen-activated protein kinase (MAPK) and the phosphoinositide-3-kinase (PI3K)/AKT signaling pathways. These pathways are two major signaling cascades and play significant roles in tumor cell proliferation, angiogenesis, migration, and survival [94].

Most FGFR–TKIs are multi-targeted inhibitors, and only a few specific/selective TKIs are in preclinical settings. PD173074 has been developed years ago and has not been approved for the treatment of cancer. Other FGFR–TKIs, including masitinib mesylate (AB1010), are multi-targeted and selectively inhibit angiogenic pathway.

### PD173074

PD173074 was developed in 1998 to stop blood vessels forming around tumors. It is a selective and potent inhibitor that binds to the TK domain of the FGFR family (Table 2). PD173074 showed (>100-fold) selective inhibition of human umbilical vein endothelial cell growth at 10 nM concentration and inhibited the formation of micro-capillaries on matrigel-coated plastic. Preclinical studies in mice have revealed that oral administration of PD173074 (25–100 mg/kg) generated dose-dependent inhibition of angiogenesis [95].

This TKI-inhibited oligodendrocyte progenitor cell proliferation has been stimulated by FGF2 [96]. It suppressed the inhibitory effects of FGF2 on pro-oligodendrocytes differentiation into oligodendrocyte in culture. Moreover, it prevented PDGF-mediated MAPK activation of oligodendrocyte progenitor cells and downregulation of myelin genes (such as CNP, MBP) and resulted in upregulation of these proteins following treatment. Collectively, data suggest that PD173074 is highly effective at selectively abolishing FGF-mediated responses of both oligodendrocyte progenitors and differentiated oligodendrocyte [96].

## 9. MET Oncogene

The MET oncogene is a heterodimer (α and β) RTK [97] and is required for the normal development [98]. MET expresses in a variety of adult tissues, but the expression is very low and restricted to cells of epithelial or mesenchymal origin [99]. The expression of MET and its ligand hepatocyte growth factor (HGF) is associated with several functions, including embryogenesis, cell proliferation, survival, differentiation, invasion, and tissue repair [100].

Deregulation of MET in cancer is due to several mechanisms, including activating mutations, gene amplification, over-expression, and increased autocrine or paracrine ligand-mediated stimulation [101]. MET over-expresses in breast cancer [102], NSCLC [103], gastric cancer [104] and several other solid tumors as well as in hematologic malignancies [105,106]. MET expression and function is associated with increased metastasis, tumor aggressiveness and poor prognosis [107].

As a MET ligand, binding of HGF/SF to the extracellular domain of MET results in receptor dimerization that induces the transphosphorylation of Tyr 1234 and 1235 residues within the kinase domain and causes a structural change within the receptor. Phosphorylation of Tyr 1349 and 1356 residues at carboxy-terminal domain are also necessary for MET signaling [108]. Mutations flank the critical Tyr 1234 and 1235-induced constitutive receptor activation in patients with hereditary papillary renal carcinoma [109]. MET oncogene uses PI3K/AKT, RAS/RAF/MEK/ERK and SRC/FAK signaling pathways for signal transduction. Activation of these pathways results in increased cell growth, proliferation, survival, motility, and angiogenesis [110].

MET has a significant role in tumor cell growth, thus providing a strong rationale for targeted therapy in cancer. Based on the structure and function of MET and its association to survival signaling pathways, there has been successful development of several MET–TKIs.

### 9.1. EMD1214063 and EMD1204831

EMD1214063 and EMD1204831 are two selective and ATP-competitive MET–TKIs that were identified recently [111]. EMD1214063 and EMD1204831 have an average IC_50_ of 3 and 9 nmol/L, respectively, when tested in the presence of recombinant human MET kinase domain and a biotinylated peptide substrate. Oral administration of EMD1214063 resulted in a strong inhibition of MET-driven tumor xenografts in mice. Several clinical trials are ongoing to check the effects of these TKIs in patients with solid tumors [112].

EMD1214063 and EMD1204831 have been noted to inhibit HGF-induced MET phosphorylation in A549 cells with an average IC_50_ of 6 and 15 nmol/L, respectively, and induced regression of human tumors in xenografted mice [111]. Both TKIs have shown similar anti-tumor effects in preclinical studies. They decreased MET autophosphorylation as well as dephosphorylation of downstream signaling proteins (AKT, ERK 1/2 and PLCγ) in cell lines with MET activation mutation [113,114].

EMD1214063 induced MET-dependent cell-cycle progression in cell lines expressing drug-sensitive forms of the MET receptor, V1238I, M1268T and H1112L MET mutated variants by 57%, 47% and 43%, respectively, and no effects in cells with MET mutant variant Y1248H and L1213V has been observed. In a xenograft tumor model mice bearing NIH3T3 cells, EMD1214063 treatment resulted in a complete regression of the sensitive H1112L mutant-derived tumors, but not in mice with L1213V tumors [113].

Inhibition of MET activity by EMD1214063 induced autophagy in gastric adenocarcinoma cell lines and inhibition of autophagy in combination with MET inhibition led to significant cell death [115]. The effects of EMD1214063 have been tested in neuroblastoma cell lines *in vitro* and *in vivo* [116]. All tested cell lines showed to be sensitive to EMD1214063 with IC_50_ values ranging from 2.4 to 8.5 nM. It induced apoptosis of cells and specifically inhibited HGF-mediated MET phosphorylation followed by MEK dephosphorylation in neuroblastoma cells. This TKI reduced neuroblastoma tumor growth in immunocompromised xenografted mice [116]. It should be considered that as these MET–TKIs are selective in nM concentrations, they might inhibit other targets with higher concentrations.

### 9.2. Tivantinib

Tivantinib (Arqule, ARQ-197) is an oral non-ATP competitive inhibitor and a staurosporine derivative that is a highly selective MET inhibitor through binding to dephosphorylated MET and preventing MET autophosphorylation (Table 2) [117]. Tivantinib was developed in 2010, and preclinical studies showed promising *in vitro* and *in vivo* results, including growth inhibition across a range of cancers [118]. Treatment of MET-expressing cancer cell lines with tivantinib inhibited cell proliferation, induction of caspase-dependent apoptosis and growth inhibition of tumor cells in xenografted mice [118].

The first clinical evaluation of tivantinib started in 2010 and the safety and tolerability, dose-limiting toxicities (DLT) and maximum tolerated dose (MTD), anti-tumor activity, pharmacokinetic, and pharmacodynamic profiles were evaluated in patients with advanced or metastatic solid tumors [110]. A maximum tolerated dose was not reached. It showed to be well tolerated and only mild to moderate toxicities were observed, including leukopenia, neutropenia, thrombocytopenia, vomiting, and dehydration. The rate of absorption was 2 to 4 h after initial dosing. Of 79 patients, 3.8% achieved a partial response and 50.6% had stable disease for a median time of 19.9 weeks [110].

Several phase II and III clinical trials are ongoing in patients with solid tumors and promising results have been achieved [119].

### 9.3. LY2801653

LY2801653 was developed in China in 2013 and is a type II MET/RON–TKI (Table 2) [120]. The mean IC_50_ value of LY2801653 for inhibition of MET autophosphorylation in HGF-stimulated H460 and S114 cells was shown to be 35.2 ± 6.9 and 59.2 nM, respectively. This TKI shows more anti-proliferative activity in cells with MET over-expression e.g., H1993 than the cells without MET gene amplification. It strongly inhibited MET phosphorylation and tumor growth in S114, U-87MG and NCI-H441 xenografted mice models [120].

This TKI inhibited NSCLC cell lines and patient-derived tumor xenografts. It prevented the constitutive activation of MET signaling and NCI-H441 cell proliferation, anchorage-independent growth, migration, and invasion. Treatment of the NCI-H441 orthotopic model significantly inhibited both primary tumor growth and metastasis. Moreover, treatment of tumor-bearing mice showed a significantly higher survival time compared to controls [121].

LY2801653 inhibited the growth of NSCLC cell lines A549, H1703 and H1993 at the IC_50_ of 627.6, 72.9 and 9.28 nmol/L, respectively. It also prevented the growth of A549 and H1993 tumor xenografts in nude mice and growth of A549-luc-C8 lung orthotopic tumor xenografts in SCID mice [122].

Currently, several specific MET–TKIs, including lSOMCL-863 [123], SOMG-833 [124], Yhhu3813 [125], and BMS-777607 [126] are under evaluation in preclinical phases.

## 10. Fms-Like Tyrosine Kinase 3 (FLT3)

FLT3 belongs to the class III RTKs and is a cytokine receptor. It expresses in several hematopoietic progenitor cells and its signaling is necessary for the normal development of hematopoietic and other progenitor cells [127]. The FLT3 gene is the most frequent gene mutation in acute myeloid leukemia (AML) and its expression is associated with a worse prognosis [128]. Several FLT3–TKIs have been developed as attractive therapeutic drugs, especially in AML patients with FLT3 mutations. The primary preclinical and clinical evaluation of the first generation of FLT3–TKIs did not show proper selectivity and pharmacokinetic properties. The second generation of these TKIs, however, has better selectivity and activity against tumor cells *in vitro* and *in vivo*. Similar to other drugs, patients have shown resistance to these TKIs. Currently, a few of them, including tandutinib, quizartinib, KW-2449 and crenolanib, have shown high selectivity.

### 10.1. Tandutinib

Tandutinib is a relatively selective FLT3–TKI (Table 2). It is a potent inhibitor of FLT3 internal tandem duplication (ITD)-transformed cell lines and human AML cell lines expressing the mutant FLT3. ITD is the most common FLT3 mutation found in up to 30% of AML patients [129].

Preliminary *in vitro* experiments showed that tandutinib inhibited wild-type FLT3 and its mutants with IC_50_ values of 30–100 nM and cell growth and proliferation were inhibited with IC_50_ values of 10–30 nM. Tandutinib has been shown to increase the survival of xenografted mice with AML tumor cells [129].

A phase I trial examined the use of tandutinib in AML patients. Data showed promising anti-tumor activity and decreased tumor cells in peripheral blood and bone marrow [130]. In a phase II study, patients with FLT3/ITD AML who were refractory, relapsed or ineligible for chemotherapy were selected for treatment [131]. Of 15 patients, two of them showed stable disease and six showed a decrease in peripheral blood and bone marrow blast cells [131].

### 10.2. Quizartinib

Quizartinib (AC220) was designed as a selective FLT3–TKI (Table 2). This TKI has been evaluated in several clinical trials. In a phase I study, quizartinib was tested in patients with relapsed or refractory AML. FLT3 phosphorylation was shown to be inhibited and of 17 FLT3/ITD AML patients, nine showed proper response [131]. In a phase II study, two cohorts of AML patients with FLT3/ITD were enrolled. In the first group, involving 92 patients with relapsed or refractory to front-line therapy showed overall response rate (ORR) in 72%. In the second cohort with 99 FLT3/ITD AML patients with relapsed or refractory to two lines of therapy ORR was 68% [132]. Results show that this TKI is highly effective against FLT3 in AML patients.

### 10.3. KW-2449

KW-2449 is a selective FLT3–TKI and is under investigation for the treatment of different cancers, including AML (Table 2) [131]. It inhibited MOLM-13 cell growth with FLT3 mutations and prevented phosphorylation of FLT3/STAT5, G1 arrest and apoptosis *in vitro* and *in vivo* in FLT3-mutated xenografted model with minimum bone marrow suppression. Furthermore, KW-2449 has been demonstrated to increase the frequency of cells in the G1 phase of the cell cycle and reduced the percentage of cells in the S phase, followed by the cell apoptosis [133].

Treatment of FLT3-mutated xenografted model displayed dose-dependent and significant tumor cell growth inhibition. Furthermore, KW-2449 showed anti-proliferative activity on primary AML cells and imatinib-resistant cells [134].

### 10.4. Crenolanib

Crenolanib is also a selective type I pan-FLT3 inhibitor with inhibitory effects on other TKs with higher concentrations (Table 2) [135]. Crenolanib has shown promising results against AML FLT3/ITD mutant isoforms, cell lines, primary human AML cells and mice models. It is strongly active against FLT3 containing ITD and D835- or F691-activating mutations and showed to inhibit the growth of MV4-11 AML cell line in xenografted mice. It has shown anti-tumor activity against Ba/F3 cells harboring FLT3/ITD mutant and MOLM-13 cells that are resistant to sorafenib both *in vitro* and *in vivo* [135].

Overall, a few selective FLT3–TKIs have been developed and have shown promising activity against leukemic cells of AML patients. More investigations in clinical trials and on the other malignancies are necessary to explore the potential anti-tumor activity of FLT3–TKIs.

## 11. TAM RTK Family: AXL and MER Inhibitors

AXL and MER RTKs belong to the TAM family, including TYRO-3, AXL and MER. The TAM receptors are defined by the presence of two Ig-like domains and two fibronectin type III repeats in the extracellular part and a cytoplasmic domain with kinase activity [136]. The vitamin K-dependent protein (Gas6) is the AXL ligand [137] and induces AXL autophosphorylation at Tyr 702 and 703 and MER autophosphorylation at Tyr 749, 753 and 754 within the activation loop [138,139]. Tyr 779, 821 and 866 in AXL and Tyr 872 in MER have been reported to be phosphorylated and provide docking sites for interaction with intracellular signaling molecules [140]. AXL is expressed in various normal organs and cells and over-expressed in several cancers [141,142]. AXL expression was found to be correlated with cancer poor prognosis [139].

Currently, among a few selective AXL–TKIs, R428 has shown encouraging results in preclinical assays and might be a proper AXL–TKI for treatment.

### R428

R428 (BGB324) is a first-in-class, highly selective AXL–TKI and has been tested in preclinical and clinical settings. R428 dephosphorylates AXL in a dose-dependent manner and prevents the invasion of both human MDA-MB-231 and murine 4T1 breast cancer cell lines *in vitro* [143].

In mice models, R428 showed acceptable plasma stability and pharmacologically proper doses have been achieved in oral administration. It inhibited breast cancer cell metastasis and angiogenesis following inhibition of AKT and ERK phosphorylation. R428 has shown proper activity in combination with cisplatin to inhibit liver and lung metastases in a breast cancer mouse model [143].

The effects of R428 have been tested on NSCLC cells *in vitro* and xenografted models, both in combination with other TKIs or chemotherapeutic agents. R428 has shown a synergistic anti-proliferation effect on NCI-H1299 (mesenchymal, EGFR wild-type, erlotinib-resistant) human NSCLC cells in combination with erlotinib. In NCI-H1299 cells xenografted mice R428 has significantly enhanced the anti-tumor activity of Docetaxel. Moreover, treatment of A549 NSCLC cell line with R428 and in combination with either erlotinib or anti-VEGF human antibody bevacizumab displayed additive anti-tumor activity. Treatment of HCC827 xenografted model with R428 has delayed the development of resistance to erlotinib [144]. R428 seems to be a good candidate for targeted cancer therapy and further development in clinical settings [143].

## 12. Targeting Receptor Tyrosine Kinase Receptor (ROR1)

ROR1 belongs to ROR–RTK family and is evolutionarily conserved between different species [15]. ROR1 consists of four extracellular parts: an Ig-like domain, cysteine-rich domain (CRD), kringle domain (KNG) and an intracellular TK domain. ROR1 over-expresses in several cancers [4,6,7,9,145] and is constitutively phosphorylated at Tyr 641, 645 and 646 within the TK domain [146,147].

There is little information about ROR1–TKIs and they are presently under intensive investigation in preclinical settings.

### IN0439365 and KAN0438063

Biochemical studies have shown that ROR1–TKIs IN0439365 and KAN0438063 are highly selective and killed leukemic CLL cells with high specificity. Among several compounds, the best (IN0439365) killed 50 times more CLL cells than normal blood lymphocytes, dephosphorylated ROR1 and inactivated PI3K/AKT/mTOR proteins [148,149]. KAN0438063 has been shown to kill CLL cells with an efficacy index value of 40. This compound induced PARP, caspase 8 and 9 cleavage and downregulated MCL-1. The selective apoptosis effect of this compound has been compared with other SMIs targeting non-ROR1 structures in CLL. This ROR1–TKI has shown to be significantly more effective (*p* ˂ 0.001) compared to PCI-32765, CAL-101, R406, R788, STK-156485 and STK-156133 inhibitors [148,149].

Similar effects were observed on pancreatic adenocarcinoma cell lines. Incubation of PaCa2 and PANC1 cells with IN0439365 induced a significant cell death following dephosphorylation of ROR1 and inactivation of SRC, PI3K, AKT, mTOR, and CREB [149,150].

Several TKIs have been developed against other oncogenic RTKs, including anaplastic lymphoma kinase (ALK), EPHA/B, IGF2R, KIT, among others (Table 1). Most of TKIs against these RTKs are not specific and more efforts are necessary to develop specific/selective RTK–TKIs.

## 13. Tumor Cell Resistance to Targeted-Cancer Therapy Agents

Resistance to the current targeted therapies, including SMIs, can be divided into the intrinsic and acquired resistance [151]. Intrinsic resistance includes the factors that exist before treatment (e.g., the presence of cancer stem cells) and acquired type develops during treatment of tumor cells which have been sensitive to the initial treatment [152]. Acquired drug resistance is caused by post-treatment changes, including alteration in drug targets, alteration in the structure and biological properties of drugs in cancer cells and activation of compensatory survival signaling pathways [153,154]. Moreover, alteration in drugs after administration, including drug inactivation, increased rates of drug efflux, changes in drug metabolism [155] and alteration of the local tumor microenvironment [156], all of which have also been identified as important factors in increasing the rate of drug resistance.

Drug resistance has been shown in various malignancies. Resistance to BCR–ABL-targeted therapies (imatinib) has been noted in AML and is associated with the intrinsic resistance of leukemic stem cells [157]. Unexpectedly, most patients with an initial good response have shown drug resistance in the first year of treatment [151]. Recent studies suggest that the long-term treatment with more than one drug (combination therapy) might reduce the abundance of cancer-associated stem cells in some patients and reduce patient relapse [151].

Dual blockade of extra- and intra-cellular parts of RTKs or targeting more than one RTK by mAbs and TKIs is likely to represent improvement in the treatment and decrease the rate of drug resistance. A combination of trastuzumab and lapatinib in xenografted mice with HER2-over-expressing cells has shown remarkable inhibition of tumor growth and survival [87]. Dual targeting of EGFR using cetuximab and gefitinib has displayed synergic effects on inhibition of tumor cell proliferation and prevented drug resistance in colon cancer cell lines [88]. A combination of trastuzumab and lapatinib has also been investigated in different trials. This dual treatment had more significant clinical activity than either agent alone *in vivo* or *in vitro* [89,90].

## 14. Conclusions

Most of the available targeted cancer therapy agents have significantly improved patients’ progression-free survival, but none of them has yet proven to cure the disease. Several RTK–TKIs and other inhibitors have been developed. Despite the considerable efforts from screening to clinical trials, which are expensive and time-consuming, only a few TKIs have entered clinical trials or have been approved by authorities for cancer treatment. Moreover, most TKIs as research tools or in the clinic are multi-targeted drugs. Multi-targeted property has several disadvantages, including side effects, a complication of the interpretation of results, and inducement of early resistance. Therefore, developing more specific/selective TKIs are necessary to overcome the current problems. On the other hand, tumor cells and their microenvironments are more complicated than in normal situations. Tumor cells simultaneously over-express several RTKs Targeting a specific RTK using a specific/selective TKI might give us a better understanding of the mechanism of tumor cell resistance. This may lead to select proper agents or prevent second-line of resistance. Therefore, identification and development of more specific SMIs will be critical for the successful treatment. To achieve this goal, several factors are required to be considered, including: (1) proper knowledge of the effects and characteristics of each TKI in preclinical studies; (2) TKIs with the highest selectivity and specificity are preferred to prevent or delay side effects and drug resistance; (3) developing more type II and III inhibitors than type I inhibitors; (4) using different animal models for preclinical *in vivo* studies; (5) a deeper understanding of the tumor heterogeneity in different individuals and the role of cancer stem cells; and (6) developing specific RTK–TKIs to target cancer stem cells. All factors are crucial to a better understanding of the nature of tumors and the raise of drug resistance.
